# Who benefited from the New Rural Cooperative Medical System in China? A case study on Anhui Province

**DOI:** 10.1186/s12913-016-1441-3

**Published:** 2016-06-05

**Authors:** Lidan Wang, Anjue Wang, Gerry FitzGerald, Lei Si, Qicheng Jiang, Dongqing Ye

**Affiliations:** School of Health Management, Anhui Medical University, No.81, Mei Shan Road, Hefei, Anhui 230032 China; School of Public Health, Anhui Medical University, No.81, Mei Shan Road, Hefei, Anhui 230032 China; Department of Development and Planning, Anhui Medical University, No.81, Mei Shan Road, Hefei, Anhui 230032 China; School of Public Health and Social Work, Queensland University of Technology, Victoria Park Road, Kelvin Grove, Brisbane, 4059 Australia

**Keywords:** Benefit incidence analysis, Concentration index, Kakwani index, Subsidy, NCMS

## Abstract

**Background:**

The goal of the New Rural Cooperative Medical System (NCMS) is to decrease the financial burden and improve the health of rural areas. The purpose of the present study is to determine how government subsidies vary between poorer and wealthier groups, especially in low-income regions in rural China.

**Methods:**

The distribution, amount, and equity of government subsidies delivered via NCMS to rural residents at different economic levels were assessed using benefit-incidence analysis, concentration index, Kakwani index, Gini index, Lorenz curve, and concentration curve. Household and health institution surveys were conducted in 2010, covering 9701 residents. Household socio-economic status, healthcare costs, out-of-pocket payments, and utilization information were collected in household interviews, and reimbursement policy was provided by institutional survey.

**Results:**

The government subsidy concentration index was −0.055 for outpatients and 0.505 for inpatients; and the outpatient and inpatient subsidy Kakwani indexes were −0.376 and 0.184, respectively. The poorest 20 % of populations received 3.4 % of the total subsidy output; while the wealthiest 20 % received 54.3 %. The results showed that the distribution of outpatient subsidies was equitable, but the hospital subsidies disproportionally benefited wealthier people.

**Conclusions:**

Wealthier people benefited more than poorer people from the NCMS in terms of inpatient and total subsidies. For outpatients, the subsidies were unrelated to ability to pay. This contradicts the common belief that the NCMS does not exacerbate benefit inequity. Long-term policy is required to tackle this problem, specifically of redesign the NCMS reimbursement system.

## Background

China has made considerable progress in its reform of public health insurance in the past few decades. The government has taken on the responsibility of financing many aspects of health insurance, especially for rural residents, who made up of 61.2 % (802 million) of China’s population as of 2013 [1]. The Cooperative Medical System was established in the 1950s. It was financed by the communes and aimed mainly at protecting rural residents. Coverage peaked in 1978 at around 90 % of the rural population. However, the system collapsed in 1979 with the disbanding of the communes, leaving most rural residents uninsured [2]. The New Rural Cooperative Medical System (NCMS) was introduced in 2003 and extended to most rural residents by 2010 with the principal goal of reduce rural people’s financial burden due to illness and to lessen the gap between rich and poor [3]. It is currently the most important type of health insurance in China with respect to protecting rural residents from poverty due to health care expenditure. Starting in 2009, the central and local governments co-financed China Yuan ¥40 per capita per annum (total ¥80 per capita per annum) and the farmers contributed ¥20 per annum in the form of NCMS premiums. In this way, the government effectively subsidized the system by 80 % [4]. The NCMS benefit package varies across China, though this mechanism of funding from central government is identical. This is because the local governments (county or city) are free to implement different reimbursement packages. Generally, the package emphases inpatient services, which are paid for according to a formula with thresholds, co-payment ratios, and ceilings. Outpatient services are reimbursed through individual medical savings accounts with fixed ceilings.

Equity is widely acknowledged to be an important policy objective in China’s health care sector [5, 6], and equity evaluation of incidence of expenditure and benefits in the health sector has attracted considerable international interest. The distribution of health care financing and expenditure in the U.S. [7, 8], Africa [9], Asia [10], and Vietnam [11] have been analyzed, and the equity of benefit of health insurance has been assessed in Tanzania [12], Iran [13], Africa [14], Nigeria [15], and Vietnam [16]. Some studies in China have analyzed the effectiveness of NCMS benefits, and results have been either inconsistent or negative results. Some of the studies have reported that NCMS can help increased outpatient service utilization at the village and township levels among people with low income [17], and reimbursement can help mitigate catastrophic health expenditure [18]. However, one study showed that participation neither improved health conditions nor increased the utilization of formal health services [19]. Another study reported that the pro-rich inequity dominated inpatient care and a pro-poor advantage was observed with outpatient care from 2007 to 2011, but the magnitude of the pro-rich inequity in inpatient expense and reimbursement decreased from 2010 to 2011 [20]. Little attention was paid to the equity of NCMS in low-income provinces.

Given that China is experiencing a widening gap between the rich and poor and that the current methods of reimbursement vary across regions in the nation, there is a clear need to determine which group is benefiting most from government subsidies [21]. This study aims to shed light on the distribution and extent of the subsidies among households of different incomes under NCMS in Anhui, and to determine who benefits the most from government reimbursement.

## Methods

### Study population

Anhui Province, located in the middle of eastern China, is one of the country’s low-income provinces. It is home to more than 61 million people. Under a multi-stage stratified random sampling method, the survey randomly selected 6 counties under NCMS representing three economic categories (wealthier, medium, and poorer). In every county, 3 townships and 9 villages were selected by economic level and geographic distribution. Then 60 households were randomly selected from each of the villages. Finally, 3149 households encompassing 9800 residents were collected for the household survey. All the members of these households who were registered local residents and who had lived in these households for a minimum of 6 months during the past year were considered eligible.

### Data collection

Data used in the study came from two sources, i.e., surveys of households and of health care institutions. Household surveys consisted of an interviewer-administered questionnaire. The structured items solicited information about demographics, economic background, health service utilization, and payment. Demographic variables included age, gender, education, employment status of household members, and number of members in the household. Economic background comprised annual income and consumption expenditures during the year prior to the survey were recorded, the latter including total expenses on food, clothing, education, traffic, housing, water, electricity, communication, fuel, other travel, medical care, entertainment, and other consumption expenditures. Health service utilization and payment included a) number of times inpatient services were received in the past year; b) type of provider (e.g., township health center, county hospital, city or higher-level hospital); c) cost of hospitalization, outpatient expenditures, and out-of-pocket payments (OOP) for inpatient care. Information about all subjects within each selected household was collected by a trained interviewer through a key individual within the household, and each household survey was conducted in 2010 by trained graduate students from the School of Health Management of Anhui Medical University.

Other data regarding NCMS policy were collected by institute-level surveys. These included a) data from the local NCMS management institutes regarding total input into the NCMS by the governments and total absolute subsidies to outpatient and inpatient care by the local NCMS; b) data from the local statistical bureaus, about economic background of sampled counties. Table 1 presents detailed data regarding descriptive and socioeconomic characteristics in the sampled counties and population. The datasets supporting the conclusions of this article is (are) included within the article.

**Table 1:** Socio-demographic features by socioeconomic level

Variables	*N* (%)				
	Poorest 20 %	Quintile 2	Quintile 3	Quintile 4	Wealthiest 20 %
Gender					
Male	940 (48.5)	944 (48.7)	949 (48.9)	955 (49.3)	939 (48.3)
Female	1000 (51.5)	994 (51.3)	992 (51.1)	984 (50.7)	1004 (51.7)
Education					
Under primary school	556 (28.7)	524 (27.0)	502 (25.9)	493 (25.4)	414 (21.3)
Primary school	729 (37.6)	718 (37.0)	686 (35.3)	664 (34.2)	577 (29.7)
Middle school	540 (27.8)	565 (29.2)	589 (30.3)	590 (30.4)	671 (34.5)
High school or more	115 (5.9)	131 (6.8)	164 (8.4)	192 (9.9)	281 (14.5)
Age (years)					
−14	367 (18.9)	362 (18.7)	340 (17.5)	342 (17.6)	241 (12.4)
15–64	1288 (66.4)	1304 (67.3)	1363 (70.2)	1366 (70.4)	1518 (78.1)
65+	285 (14.7)	272 (14.0)	238 (12.3)	231 (11.9)	184 (9.5)

### Statistic analysis

Benefit-incidence analysis (BIA) has been used extensively to investigate the equity of NCMS benefit in the study. The benefit incidence is the subsidies that the government pays to rural residents. The distribution of benefit incidence was here analyzed in relation to economic status of the beneficiaries in terms of their adult equivalent consumption expenditure (AECE). In this study, the “interest in BIA” is how subsidies for outpatient and inpatient care from NCMS vary with 5 quintiles. BIA basically involves five steps [22–25]: (1) Calculation of the AECE of rural residents on the basis of household surveys; (2) aggregation of the sample of rural residents into five quintiles based on AECE assessment; (3) calculation of the value of the benefit to rural residents from the government; (4) calculation of the relative benefit for different quintiles; (5) calculation of the index for the distribution of fairness.

Taking household size and composition of rural area into account, the value of AECE here served as an indicator of household standard of living. AECE was here defined as annual consumption expenditure per adult equivalent in a household, and the number of adult equivalents (AE) was defined as follows:

$$ \mathrm{A}\mathrm{E} = {\left(\mathrm{A} + 0.5\ \mathrm{K}\right)}^{0.75} $$

Here, A is the number of adults in the household and K is the number of children (0–14 years old). Residents were divided into five quintiles according to AECE. Quintile 1 was the poorest 20 % of the population, and quintile 5 was the wealthiest 20 %.

The ceiling reimbursement in individual accounts was set as the value of the outpatient subsidy for all individuals who received and paid more than the ceiling on outpatient services. The inpatient health care subsidy was considered the difference between total inpatient cost and individual OOP.

**Fig. 1:**
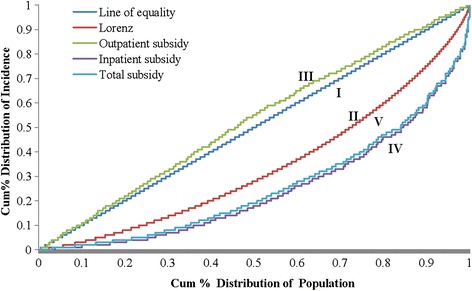
Inpatient utilization of health resource among rural residents in Anhui, 2009

The Lorenz curve (LC), concentration curve (CC), concentration index (CI) and Kakwani index (KI) have been widely used in the analysis of equity of government subsidies and use of public services [15]. The LC is often used to represent distribution of wealth or assets. It shows what percentage (y %) of the total wealth is possessed the bottom x % of a population. In the study, cumulative percentage of the population was plotted on the x-axis by ascending AECE, and the cumulative percentage of annual AECE was plotted on the y-axis. The CC for subsidy shows the cumulative percentage of NCMS subsidies for the sampled population against the cumulative percentage of population, ranked by AECE. The equity of subsidy is measured by distance between CC and LC. If p% populations had exactly p% subsidies (consumption expenditure), the CC of subsidy (LC) would lie along the 45° line, in which case the distribution of subsidy (consumption expenditure) would be absolute equity. When the population had the same subsidy relative to their consumption expenditure, the CC of the subsidy would match the LC for consumption expenditure, which means that the distribution of subsidy would be absolute equity. If the subsidy’s CC lay above the LC, which indicates that poorer individuals shouldered a greater than average portion of consumption expenditure. In this case, the NCMS subsidy is deemed to prior to poor. When the wealthier residents benefit more (pre-rich), if the subsidy’s CC lay below the LC. In this way, there were 4 curves in the present study. Curve I was the absolute equity line (the 45° line), Curve II is the LC, Curve III is the outpatient benefit concentration curve, Curve IV is the total benefit concentration curve, and Curve V is the inpatient benefit concentration curve (Fig. 1).

In this way, the CI and KI of NCMS subsidies were calculated to facilitate analysis of the degree of absolute and relative equity, respectively. CI is here defined as the distribution of health subsidy contributions across the population ranked by AECE. CI was calculated as follows:

$$ CI = 2*\  area\  between\  the\ 45o\  line\  and\ CC\  of\  health\  subsidy $$

The value of CI ranges from −1 to 1. KI is defined as twice the area between the LC for consumption expenditure and the CC for subsidy, with a range of (−2, 1). KI is calculated using the difference between the CI and Gini coefficient, and the latter is computed by payments spent on consumables per capita against AECE. A positive value of CI and KI indicates that wealthier individuals benefit more than poorer individuals, so that the LC for consumption expenditure lies above the CC for subsidy. Negative values indicate that poorer individuals benefit more, and the LC lies below the CC. The value of the indexes is zero if the subsidy is proportional. KI facilitates comparison of the level of relative equity at different times and in different areas without considering different social-economic contexts, which is why economic status and health subsidy factors must be included in the model [26]. In this way, the differences in KI across different regions and years can be used to assess the level of inequity between different areas and over time.

### Subject protection

Ethical approval was obtained from Anhui Medical University Research Ethics Committee prior to data collection. The interviewees were required and sign a consent form after being read an introduction to this study by the interviewers.

## Results

### Features of sampled residents

The socio-demographic features of sampled rural residents are shown in Table 1. Among all of the sampled rural residents, 48.6 % were female, 25.7 % did not complete primary school, and more than half of them had only elementary school education. As expected, more individuals with higher education were observed in wealthier groups. More children (individuals younger than 14 years old) and elderly (older than 65 years old) were found in poorer quintiles (Table 1). In terms of sampled areas, Dangtu showed the best economic status, with about U.S. $774 (U.S. $1.00 = ¥6.831) total consumption expenditure per capita annually, and the other five counties had lower expenditures per capita (Table 2).

**Table 2:** Benefits of inpatient services under NCMS in sampled counties

Indices	Feixi	Huaining	Qingyang	Lujiang	Dangtu	Huoqiu	Average of Anhui
Expenditure per capita (US $)^a^	427	325	507	399	774	390	535
Rate of participation in NCMS (%)	96.7	99.6	98.5	93.4	96.4	93.6	93.6
Hospitalization rate in NCMS (%)	6.0	6.0	6.1	6.1	5.7	5.9	6.6
Benefit episodes of inpatients (thousand)	42.3	34.1	13.5	57.4	30.6	37.0	3,045.7
Average benefit per inpatients service ($)^a^	236.0	259.2	238.5	222.3	280.3	451.3	217.3

*Notes:* ^a^- United States dollars, based on the currency exchange rates of 6.831 China Yuan to US $ 1.00 in 2009

### NCMS in the target area

The average NCMS participation rate in the counties was relatively high in 2009 (>93 %) than in other years. The participation rate in Huaining was the highest, at 99.6 % and Lujiang had the lowest rate at 93.4 % (Table 2). In 2009, the total funding available from NCMS in Anhui was U.S. $691 million, of which 40.4 % was from the central government, 29.7 % from provincial governments, and 10.2 % from city and county funds. Rural residents contributed 19.7 % from interest on investments and others. However the NCMS expenditure in the same year was U.S. $742 million; 107.4 % of that year’s revenue, and 85.8 % of the total fund pool.

### Service utilization

As shown in Table 2, the hospitalization rates of NCMS enrollees in sampled counties were lower than the average rate for both urban and rural residents in Anhui. Lujiang benefited most in terms of inpatient services (57.4 thousand patients in total) and in terms of hospitalization rate (6.1 %, the highest of all sampled counties). The average reimbursement for per inpatient service was the highest ($451.3) in Huoqiu, which also had a lowest hospitalization rate (5.9 %). In terms of 5 quintiles, the highest utilization of both outpatient and inpatient was observed in wealthiest quintile (52.5 % and 10.1, respectively), and the lowest percentage was observed in the poorest group (Table 3). Wealthier groups also showed more absolute benefit per inpatient service, with lower proportions of their expenditures reimbursed. In other words, the health expenditures were higher in wealthier groups than in poorer groups. The expenditures of the wealthiest quintile were about 7 times higher than that of the poorest quintile.

**Table 3:** Use and benefits by socio-economic level

Quintiles	Use		Benefits	
	Outpatient services (%)	Inpatient services (%)	Average benefit per inpatient service (US $)	Proportion of benefit (%)
Poorest 20 %	46.0	2.7	64.9	41.2
Quintile 2 (%)	49.1	4.6	108.8	41.4
Quintile 3 (%)	49.4	7.0	127.6	39.7
Quintile 4 (%)	52.2	7.3	158.1	33.3
Wealthiest 20 %	52.5	10.1	350.0	29.7

**Fig. 2:**
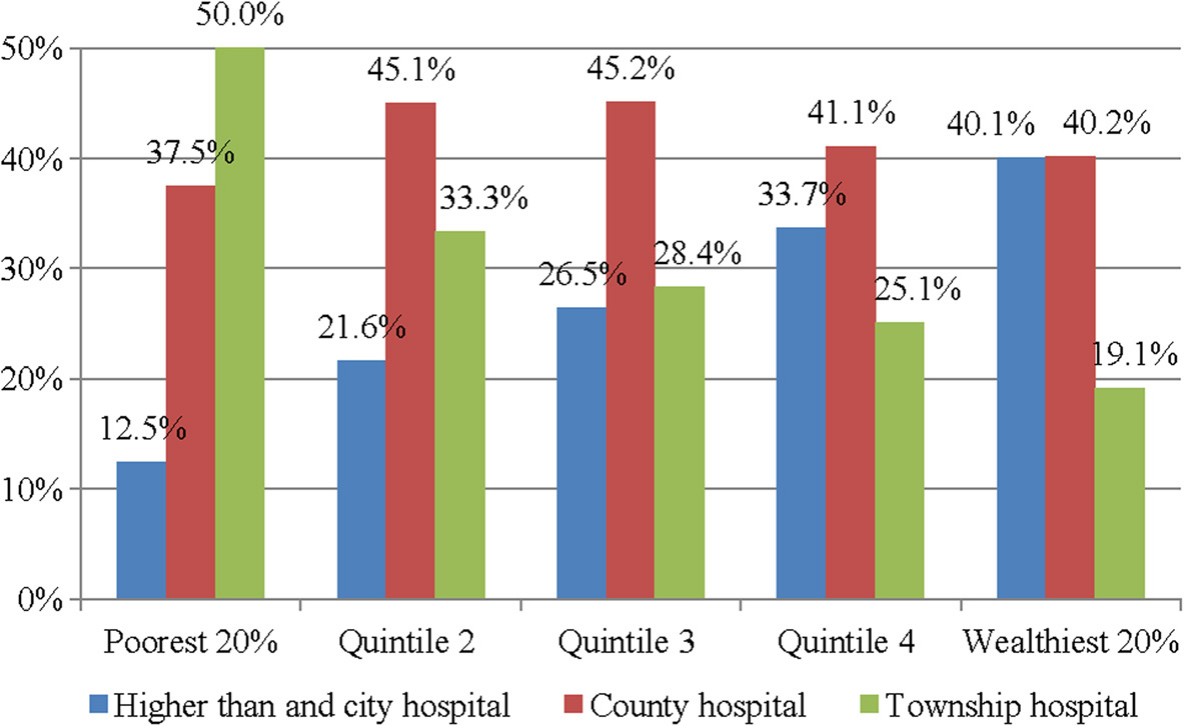
Expenditures and beneficiaries of the NCMS fund in Anhui, 2009

Over 30.5 % of all inpatients chose hospitals at the city level or higher, and 42.0 % chose the county hospitals while only 27.69 % chose township hospitals. The majority of the poorest quintile selected county and township hospitals, accounting for 87.5 % of the total hospitalization utilization by this quintile. However, most of the inpatients in the wealthiest quintile chose county, city-level, or higher-level hospitals, accounting for 80.9 % of the total for this quintile. In general, most of the total beneficiaries in middle groups (quintile 2, 3, 4) chose county hospitals (Fig. 2).

**Fig. 3:**
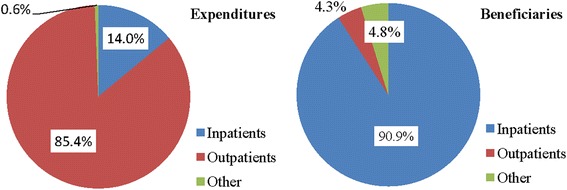
Lorenz curve and concentration curve of NCMS benefits in Anhui, 2009

Under the reimbursement package of Anhui NCMS in 2009, the reimbursement for outpatients from individual accounts was $2.9, and the deductible for inpatient services provided by township health centers, county hospitals, and hospitals at higher levels was about $15, $29–44, and $73–88, respectively; the co-payments for inpatient services from these categories of providers accounted for 70 % for township health centers, 65–70 % for county hospitals, and 50–65 % for hospitals at the city level or higher, with a ceiling of $732. In terms of service activities, 3.63 million episodes of care were subsidized, consisting of 0.51 million (14.0 %) inpatient and 3.10 million (85.4 %) outpatient visits. In terms of expenditure, 89.2 % (U.S. $662) was paid for inpatient services and only 4.2 % for outpatient care (U.S. $31). The ratios are given in Fig. 3.

### Benefit incidence for different population groups

Table 4 shows that the equivalent expenditure of the poorest 20 % households made up 7.8 % of the total expenditures; and that of the wealthiest 20 % households made up 40.2 %. More services were observed in wealthier group, especially the wealthiest 20 % of the population, who took 31.9 % inpatients (as 3.7 times as the person-time of poorest group). In terms of benefits, a higher proportion of outpatient subsidies were observed in poorer groups, although the difference had no statistically significant. However, the poorest 20 % of the population received 3.4 % of the total subsidies and the wealthiest 20 % population received 54.3 %. The wealthiest quintile consumed a larger share of inpatient healthcare resources. The pattern of distribution of total benefits was similar to that of inpatient benefits.

**Table 4:** Distribution of the use and benefits by socio-economic level

Quintiles	AECE	Use		Benefits		
		Outpatients	Inpatients	Outpatients	Inpatients	Total
Poorest 20 %	7.8	18.5	8.6	21.7	3.4	4.2
Quintile 2 (%)	12.3	19.7	14.6	21.9	8.3	8.9
Quintile 3 (%)	16.7	19.8	22.0	21.5	14.6	14.9
Quintile 4 (%)	23.0	20.9	22.9	17.9	19.5	19.5
Wealthiest 20 %	40.2	21.1	31.9	17.0	54.3	52.6
CI	0.321	0.041	0.078	−0.055	0.505	0.480
KI	N/A^a^			−0.376	0.184	0.159

*Note:* ^a^- not applicable

CI and KI reflected the inequity more clearly. The value of CI for AECE was 0.321, which was also called the Gini coefficient. CI of utilization for outpatients (0.041) and inpatients (0.078) was positive and close to zero. The CI of outpatient benefit was estimated to be −0.055; and the CIs of inpatient and total benefits were estimated to be 0.505 and 0.480. The KI of outpatient benefits was −0.376; but the KIs of inpatient (0.185) and total benefits (0.16) were positive too. As manifested by the negative value of CI for outpatient, the Curve III (CC for outpatient) that lies above the 45°line (Fig. 3). Conversely, the CCs for inpatient and total benefit distribution were below the LC for consumption expenditure. That is consistent with the positive value of KI for inpatient and total benefit.

## Discussion

The present study showed that the amount spent on inpatient health care increased with consumption expenditure and with increased service utilization. Wealthier individuals were more likely to choose higher-level hospitals and then see a lower rate of reimbursement, which is consistent with previous studies showing that outpatient and inpatient services at county-level hospitals were disproportionately utilized by people with higher incomes [17]. Although poorer individuals were likely to receive benefits more often than richer ones, wealthier individuals received more money. This at least partially confirms that every individual subsidy payments to poorer individuals are lower than to those in higher consumption expenditure groups. That may be explained by the fact wealthier individuals can afford higher prices and higher-level health care services because of their greater ability to pay. As shown here, wealthier individuals were hospitalized more often, included a higher proportion of working-age individuals, and prefer higher-level health services (i.e., county, province, or higher level medical institutes) (Fig. 2). This is consistent with the findings of previous studies [27, 28].

The location of the CC curves for benefit relative to LC for consumption and the values of CI and KI showed that outpatient benefits made a slight reduction in the rich-poor gap but inpatient benefits did not. The limited impact on outpatient subsidies can probably be explained at least in part by the design of the benefit package. As a result of limited financing, many outpatient services in many areas are not covered or are only partially covered by NCMS. However, previous studies have focused primarily on inpatient services with relatively high reimbursement rates because they are the main causes of catastrophic health care expenditure for households [29]. In this way, NCMS members preferred inpatient services whenever possible. One previous study in China showed that the NCMS had no or little impact on the utilization of outpatient services, but it was associated with higher utilization of inpatient services [30]. These findings were realized by policy-making, and most counties are currently trying to cover or increase the reimbursement rate of outpatient services in the NCMS benefit package.

Taking the absolute equity into account, benefit incidence should be equal across all quintiles. However, the present study showed that the wealthier quintiles received less in outpatient benefits and more inpatient and overall subsidies; which suggested that the NCMS outpatient subsidy was slightly in favor of the poor and that wealthier rural residents benefited more from inpatient and total subsidies. This is opposite to the intention of NCMS. These findings contradict the common belief that NCMS helps to narrowing the gap between rich and poor. These findings are supported by a study of 10 counties in China [29]. According to the NCMS subsidy policies regarding deductibles, co-payment rates, and ceilings, the patients only benefit from NCMS after they have obtained health services and spent a sum on medical treatment between the deductible and ceiling. When faced with economic limitations, poor residents cut down on health care. Even worse, the poorest are likely to be excluded from inpatient treatment because they are unable to pay for the threshold. For them, even small health care expenditures can be a catastrophic shock to their household budgets. Some studies have discussed the economic burdens incurred by individuals and household due to high OOP on health care [18, 31]. Poorer residents are also less likely than wealthier people to be able afford large co-payments, especially when they have to pay the whole fee before they can receive reimbursement for any part of it. Individuals with a greater ability to pay are more likely to receive NCMS subsidies [32].

Given the current state of the NCMS, the narrow beneficiary scope and low reimbursement rate may be viewed as the main reasons for the differences in health service utilization, especially by the poor [30]. The reimbursement package must be redesigned to secure access to universal health care at an affordable price. Some researchers have argued that the other forms of public insurance should be used to adjust the reimbursement model in order to improve health service utilization by the poorest families [33]. The results of the present study strengthen the suggestion that NCMS benefit package should remove the deductible and ceiling, increase the co-payment ratio, raise the reimbursement rate, and broaden the scope of NCMS subsidies [34], especially for disadvantaged groups with chronic or catastrophic illness or low income. Based on the findings of the present study, expanding outpatient savings account may encourage the poorest households seek health services and so reduce inpatient service utilization in the long run.

### Limitations

The study has some limitations. First, the study analyzes the distribution of the NCMS subsidy in Anhui Province. The benefit packages vary across regions, and therefore may not be readily generalizable to other parts of China. Second, the data were collected by survey and therefore dependent on the reliability of each respondent’s memory. Last, the relationship between health service utilization and inequity benefits are not expressed directly in this study.

## Conclusion

The findings suggest that the amount spent on inpatient health care and the absolute values of inpatient subsidies are greater in wealthier groups under the current funding arrangements. However, for outpatient subsidies, the benefits were spread more evenly across each quintile. As a result, the intention of the NCMS to reduce the gap between rich and poor population is having a perverse effect. Long-term policy is required to tackle this problem, especially the design of the NCMS’s reimbursement system.

## Abbreviations

BIA, benefit-incidence analysis; CC, concentration curve; CI, concentration index; KI, Kakwani index; LC, Lorenz curve; NCMS, new rural cooperative medical system.
